# A rare case of lymphadenitis and pulmonary disease caused by *Mycobacterium paraffinicum*

**DOI:** 10.1002/rcr2.414

**Published:** 2019-03-12

**Authors:** Pei Sze Carmen Tan, Ruad Perera

**Affiliations:** ^1^ Department of Respiratory and Critical Care Medicine Tan Tock Seng Hospital Singapore; ^2^ Department of Respiratory Medicine Royal Perth Hospital Perth Western Australia Australia

**Keywords:** Lymphadenitis, non‐tuberculous, pulmonary infection, rare

## Abstract

With over 150 species, non‐tuberculous mycobacteria are increasingly recognized to be important human pathogens that pose diagnostic and management challenges. We report a rare case of cervical lymphadenitis and pulmonary disease caused by *Mycobacterium paraffinicum* in a 64‐year‐old man who presented with three‐month history of increasing right‐sided painless neck lump. His medical history included rheumatoid arthritis, which was managed with leflunomide and methotrexate. Computed tomography scans of his neck and thorax revealed a right lower neck and supraclavicular fossa cystic mass with peripheral enhancement and bilateral multiple small pulmonary nodules. *M. paraffinicum* was cultured from a fine‐needle aspiration of the mass. Two out of three sputa were acid‐fast bacilli smear positive but cultures did not yield any viable organism. He developed spontaneous discharge of purulent material via a sinus, which drained over two months and recovered with a completely healed sinus without any further treatment.

## Introduction

Non‐tuberculous mycobacteria (NTM) is known to cause four clinical syndromes in humans: progressive pulmonary disease, superficial lymphadenitis, disseminated disease, and skin/soft tissue infection. Over 150 species have been described and the number continues to increase with improved isolation and identification techniques 1. *Mycobacterium paraffinicum* was first isolated in soil in 1956 but only achieved species status in 2010 2. To date, little is known about its pathogenic potential, drug susceptibility profile, and treatment outcome. We report a case of lymphadenitis and pulmonary disease caused by *M. paraffinicum*.

## Case Report

**Figure 1:**
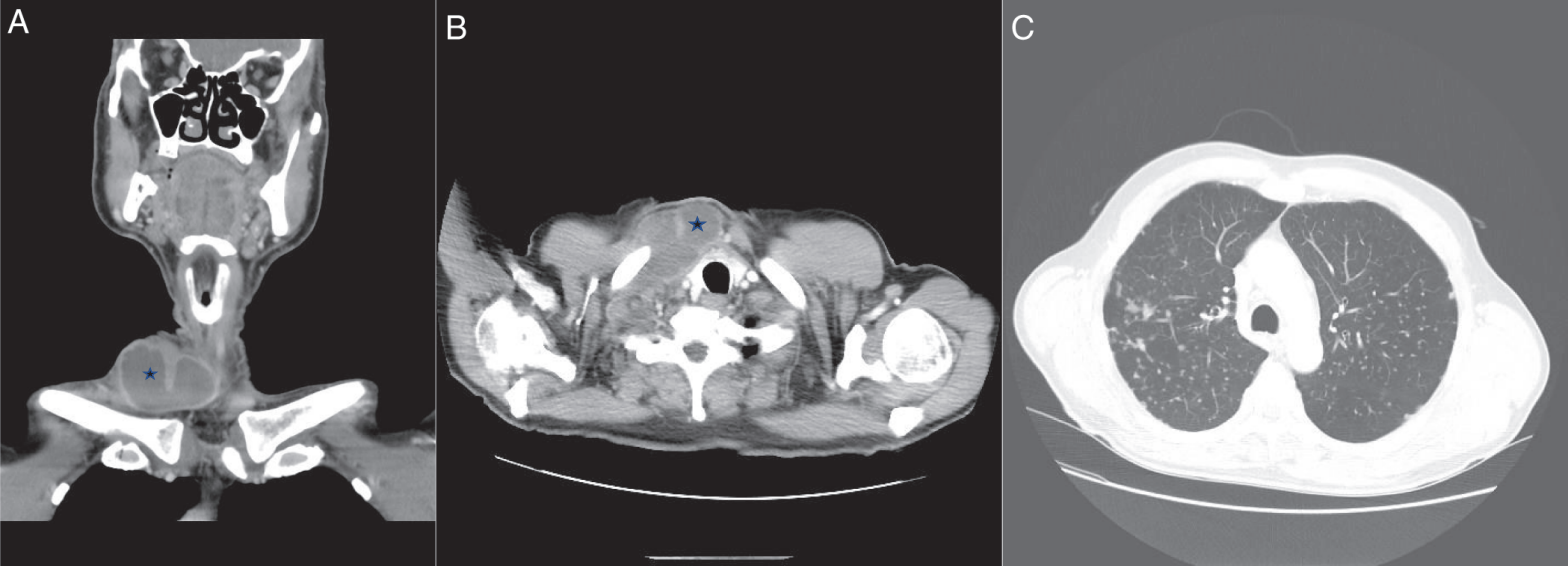
(A,B) Axial and coronal computed tomography (CT) scans of right lower neck cystic mass with peripheral enhancement. (C) CT thorax showing multiple well‐defined pulmonary nodules in bilateral upper lobe predominance.

**Figure 2:**
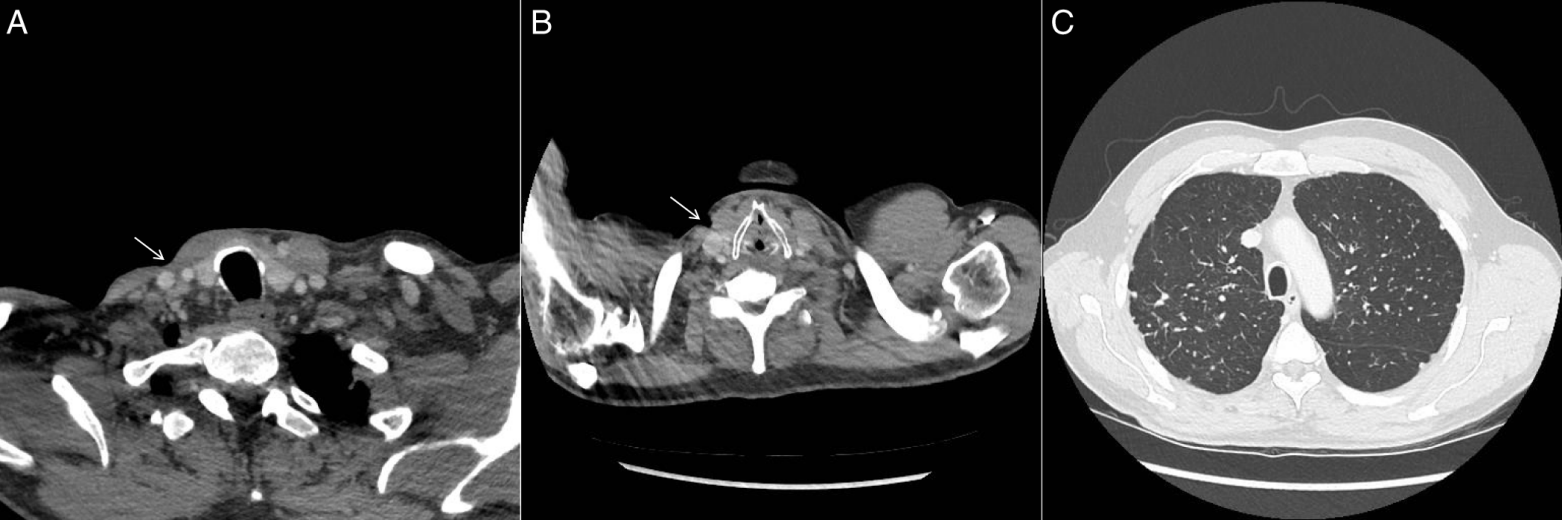
(A,B) Axial computed tomography (CT) scans showing complete resolution of necrotic nodal mass and healing sinus (white arrow) at six and 12 months, respectively. (C) CT thorax at 12‐month follow‐up showing stable pulmonary nodules.

A 64‐year‐old man with three‐month history of increasing right‐sided neck lump was reviewed at the local tuberculosis control centre for suspected tuberculosis. Three weeks prior, he underwent an ultrasound‐guided fine‐needle aspiration (FNA) of the lesion arranged by his general practitioner, which yielded 20 mL of purulent material. Computed tomography (CT) scan of his neck and thorax (Fig. 1A–C) revealed an enhancing mass measuring 85 mm × 44 mm × 57 mm within the lower neck and supraclavicular fossa and multiple parenchymal nodules over bilateral upper lobes. The patient denied any fever, night sweats, or weight loss. Except for a mild chronic cough, systemic review was unremarkable. There was no prior history of diabetes mellitus, recent travel, or history of trauma/open wounds on his neck. He had a 10‐year history of rheumatoid arthritis, which was controlled with leflunomide 10 mg daily and methotrexate 20 mg weekly. He is an active smoker of 20 pack‐years. He worked as a foundry metal machine operator all his life. Clinically, the patient was afebrile, normotensive, and appeared well. The right cervical mass was firm, non‐tender, and with no overlying skin changes. A small sinus just above the supraclavicular fossa oozing greenish creamy fluid was seen. This spontaneous discharge had developed two weeks after FNA. Basic blood tests were normal; white cell count 6.09 × 10^9^/L, haemoglobin 131 g/L, platelet 243 × 10^9^/L, C‐reactive protein 4.9 mg/L, Na 137 mmol/L, K 4.7 mmol/L, Ur 8.2 mmol/L, Cr 103 μmol/L, albumin 39 g/L, haemoglobin A1c 5.5%, and human immunodeficiency virus screening test negative. The FNA specimen was acid‐fast bacilli (AFB) smear 2+ and GeneXpert MTB/RIF molecular assay was negative for *Mycobacterium tuberculosis* complex. *M. paraffinicum* was identified based on 99% homology with 16S rRNA gene sequence information. The minimum inhibitory concentrations (MIC) of antibiotics were as follows: clarithromycin MIC: 8.0 μg/mL; rifabutin MIC: 1.0 μg/mL; ethambutol MIC: 16.0 μg/mL; isoniazid MIC: >8.0 μg/mL; moxifloxacin MIC: 4.0 μg/mL; rifampin MIC: >8.0 μg/mL; trimethoprim/sulfamethoxazole MIC: >8/152 μg/mL; amikacin MIC: 16.0 μg/mL; linezolid MIC: 32.0 μg/mL; ciprofloxacin MIC: 8.0 μg/mL; and streptomycin MIC: 16.0 μg/mL. Two out of three sputa were AFB smear positive, but cultures failed to yield any viable organism. Cytology showed no malignant cells but numerous macrophages and neutrophils. Spontaneous discharge of the neck lump persisted over a two‐month period and was managed with regular wound care. A multidisciplinary decision was initially made to treat residual disease with rifabutin, clarithromycin, and moxifloxacin. However, he went on to recover with a completely healed sinus before any drug therapy was started. Follow‐up CT scans at six and 12 months (Fig. 2A–C) confirmed resolution of the neck mass and stable pulmonary changes. His lung function tests on follow‐up showed only mild obstruction of forced expiratory volume in 1 s 2.30 L (79%) and normal diffusion capacity. Plans for further treatment were held off. He has since remained well and is maintained on close monitoring.

## Discussion

*M. paraffinicum* is a slow‐growing mycobacterium and a rare cause of clinical NTM infection, with only two case reports in published literature to date 3, 4. Both were pulmonary infections in elderly females with advanced bronchiectasis at presentation and treated with similar antimicrobials (azithromycin, ciprofloxacin, and linezolid). Both treatments were prematurely discontinued due to intolerable gastric adverse effects.

In comparison, our patient was a middle‐aged man with chronic immunosuppression secondary to underlying rheumatoid arthritis and immunosuppressive therapy who was diagnosed with *M. paraffinicum* cervical lymphadenitis and pulmonary disease. Even though his sputum cultures were negative, repeated positive AFB smears and radiological findings were suspicious for concomitant pulmonary involvement.

A 10‐year review (*n* = 15) reported 14 (93%) adults with NTM lymphadenitis 5. Most presented with multiple lymphadenitis positive for rapid‐growing mycobacteria, with *Mycobacterium abscessus* being the commonest. Like our patient, one third of the cohort had underlying clinical immunodeficiency. Management of adult NTM lymphadenitis was variable with two to three antibiotics and/or surgical resection 5. Treatment challenge is further compounded by limited data on in vitro susceptibility as well as paucity of clinical experience in empirical therapeutic regimens and treatment outcomes 4.

We add to literature the first case of *M. paraffinicum* causing lymphadenitis, which resolved with spontaneous discharge. Further follow‐up is essential to monitor for relapse and progressive pulmonary disease.

### Disclosure Statement

Appropriate written informed consent was obtained for publication of this case report and accompanying images.
